# Biologically controlled synthesis and assembly of magnetite nanoparticles†
†Electronic supplementary information (ESI) available. See DOI: 10.1039/c4fd00240g



**DOI:** 10.1039/c4fd00240g

**Published:** 2015-05-01

**Authors:** Mathieu Bennet, Luca Bertinetti, Robert K. Neely, Andreas Schertel, André Körnig, Cristina Flors, Frank D. Müller, Dirk Schüler, Stefan Klumpp, Damien Faivre

**Affiliations:** a Department of Biomaterials , Max Planck Institute of Colloids and Interfaces , Science Park Golm , 14424 Potsdam , Germany . Email: damien.faivre@mpikg.mpg.de; b The University of Birmingham , School of Chemistry , Edgbaston , Birmingham , B15 2TT , UK; c Carl Zeiss Microscopy GmbH , Training , Application and Support Center , Carl-Zeiss-Str. 22 , 73447 Oberkochen , Germany; d Madrid Institute for Advanced Studies in Nanoscience (IMDEA Nanociencia) , C/Faraday 9 , Madrid 28049 , Spain; e Universität Bayreuth , Lehrstuhl für Mikrobiologie , Universitätssstraße 30 , 95447 Bayreuth , Germany; f Department of Theory and Biosystems , Max Planck Institute of Colloids and Interfaces , Science Park Golm , 14424 Potsdam , Germany

## Abstract

Magnetite nanoparticles have size- and shape-dependent magnetic properties. In addition, assemblies of magnetite nanoparticles forming one-dimensional nanostructures have magnetic properties distinct from zero-dimensional or non-organized materials due to strong uniaxial shape anisotropy. However, assemblies of free-standing magnetic nanoparticles tend to collapse and form closed-ring structures rather than chains in order to minimize their energy. Magnetotactic bacteria, ubiquitous microorganisms, have the capability to mineralize magnetite nanoparticles, the so-called magnetosomes, and to direct their assembly in stable chains *via* biological macromolecules. In this contribution, the synthesis and assembly of biological magnetite to obtain functional magnetic dipoles in magnetotactic bacteria are presented, with a focus on the assembly. We present tomographic reconstructions based on cryo-FIB sectioning and SEM imaging of a magnetotactic bacterium to exemplify that the magnetosome chain is indeed a paradigm of a 1D magnetic nanostructure, based on the assembly of several individual particles. We show that the biological forces are a major player in the formation of the magnetosome chain. Finally, we demonstrate by super resolution fluorescence microscopy that MamK, a protein of the actin family necessary to form the chain backbone in the bacteria, forms a bundle of filaments that are not only found in the vicinity of the magnetosome chain but are widespread within the cytoplasm, illustrating the dynamic localization of the protein within the cells. These very simple microorganisms have thus much to teach us with regards to controlling the design of functional 1D magnetic nanoassembly.

## 1. Introduction

One of the central principles of nanoscience is that the physical properties of nanoparticles are size-dependent. Recent attention in the field has focussed on the assembly of nanoparticles, since new physical properties can emerge as a result of this organization.^1^ However, whilst many chemical approaches have been developed to control a nanoparticle's dimension or morphology, methods to control their organization have remained scarce.^2^

**Fig. 1:**
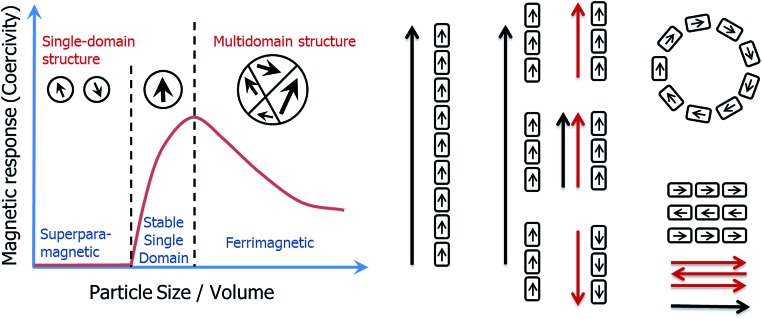
Scheme of the magnetic properties of individual magnetite nanoparticles (left) and their assembly (right). Magnetotactic bacteria are able to form magnetosomes of dimensions maximizing their magnetic properties (stable single domain) in a chain organization, which also maximizes their potential to be used as a compass by the cell.

The iron oxide magnetite (Fe_3_O_4_) represents the archetype of a nanoparticle as described above. Magnetic properties are indeed size dependent in the nm size-range (Fig. 1), with particles smaller than about 30 nm being superparamagnetic (SP, no permanent magnetic signal at room temperature in the absence of an external field), particles larger than 30 nm but smaller than 100 nm being stable single domain (SSD, one domain, remanent magnetization), and particles larger than 100 nm being multidomain (MD, more than one domain, remanent but reduced volume magnetization when compared to SSD).^3,4^ In addition, the magnetic properties are affected by the morphology of the nanoparticles,^5^ their oxidation state,^6^ and by their organization.^7,8^

**Fig. 2:**
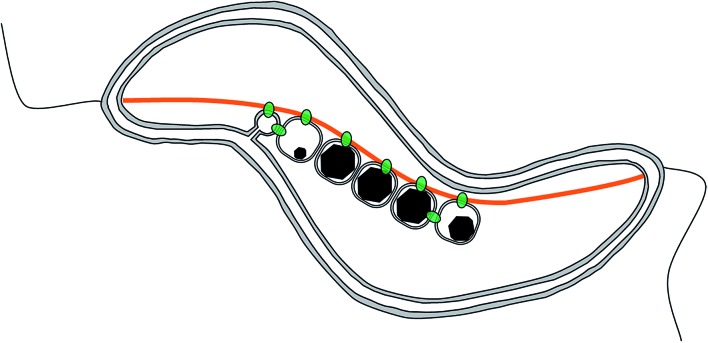
Scheme of a magnetosome chain in a magnetotactic bacterium. The 2 main molecular players are depicted as follows: in orange, MamK forms a filament spanning from one cell pole to the other. In green, MamJ attaches the magnetosome to the filament. However, the clear localization of both proteins is not clear.

Magnetotactic bacteria are ideal candidates for studying the synthesis and organization of magnetite nanoparticles. These microorganisms indeed synthesize magnetic nanoparticles called magnetosomes that are membrane-enclosed crystals made of magnetite or greigite (Fe_3_S_4_, the iron sulphide equivalent to magnetite).^9^ These nanoparticles are aligned in chain to form a single magnetic dipole strong enough to possibly passively orient the cell along the Earth magnetic field lines, to help the organisms find their preferred habitat.^10,11^ The chain is a hierarchically-structured material made from the assembly of nanoparticles, for which the mineralogy,^12^ the dimension,^13–18^ and the crystal orientation^19^ are the results of an interplay between physical processes,^20–23^ mostly based on magnetic interactions, and biological control exerted by the bacteria based on its genetic programme.^24–30^ In particular, 2 proteins have been highlighted for the role they play in the formation of the magnetosome chain (Fig. 2): MamJ, which is only found in magnetospirilla, has been described as the magnetosome connector, which enables the binding of the magnetosome particles to the magnetosome filament, likely made from polymeric subunits of MamK. The *mamK* gene, which is found in the genome of all sequenced magnetotactic bacteria, is a member of the actin family and is involved in the building of a backbone to which the magnetosome can attach. The MamK protein forms long bundles of filaments *in vitro*.^31–34^ *In vivo*, the cells also form long filamentous structures that span the long axis of the cells from cell pole to cell pole, as shown by cryo-electron tomography.^25–27^ Tagging MamK with fluorescent markers and studying the localization of the protein by fluorescence microscopy has shown that MamK is at least involved in the building of the filamentous structure *in vivo*.^24,25^ However, the exact distribution of MamK *in vivo* could not be fully clarified, since only a very limited amount of samples have been analysed by cryo-electron tomography where in addition the nature of the filament cannot be granted, and since images obtained by optical fluorescence microscopy do not exhibit a resolution that is sufficient to study this point.

MamJ and MamK were shown to interact *in vivo* ^28,29^ and the connection between MamJ and MamK is mechanically extremely stable.^35^ The exact nature of the MamJ–MamK interaction has not yet been elucidated. Evidence for direct interaction of MamJ and MamK comes from two-hybrid assays as well as FRET-experiments.^28,29^ In addition, mutants in either *mamK* or *mamJ* have no magnetite crystallization defect but display severe magnetosome alignment perturbations. For example, deletion of the *mamJ* gene abolishes formation of a magnetosome chain completely and clusters of magnetosomes are observed within the cells^27,28^ whereas *mamK* is essential to form a coherent and properly positioned chain in MSR-1, suggesting that both proteins act in the same cellular process of magnetosome positioning.^25^ In addition, in the related organism *Magnetospirillum magnetotacticum* AMB-1, the protein filaments formed by MamK subunits have been shown to be dynamic, and these dynamics depend on MamJ.^24,31^ Mutants with non-dynamic filaments are impaired in magnetosome chain formation as well. Together, these observations suggest a model in which MamK forms a filament that spans the long axis of the bacterial cell. MamJ anchors the magnetosomes to this filament and may regulate MamK dynamics, which is needed for formation of a coherent magnetosome chain and its proper positioning within the cell.

In this communication, we therefore characterize the magnetosome chain by cryo-focused ion beam (cryo-FIB) slicing and scanning electron microscopy (SEM) imaging and confirm the high degree of alignment typically observed for magnetosome synthesized by the wild-type cells of *Magnetospirillum gryphiswaldense* MSR-1. In addition, we show by super resolution optical microscopy that MamK filaments are widely dispersed within the cytoplasm of the cell and therefore their localization is not limited to the magnetosome chain, as shown so far.

## 2. Materials and methods

### (a) Cryo-FIB imaging and image reconstruction

#### Sample preparation

Magnetotactic bacteria (*Magnetospirillum gryphiswaldense*) were frozen under high pressure using a Leica HPM100. The samples were mounted on a cryo sample holder in the preparation box of the VCT100 shuttle system (Leica Microsystems, Vienna, Austria) at the temperature of liquid nitrogen. By using the VCT100 shuttle, the sample holder was transferred to the SCD500 sputter coater (Leica Micro-systems, Vienna, Austria) at –154 °C cryo-stage temperature and 4 × 10^–6^ mbar chamber pressure. The sample was sputter-coated with a 6 nm platinum layer at 0.06 nm s^–1^. During sputter coating an argon pressure of 2 × 10^–2^ mbar was used. After coating, the sample was transferred to the Auriga60 CrossbeamR system (Carl Zeiss Microscopy GmbH, Oberkochen, Germany), using the VCT100 shuttle at a cryo-stage temperature of –154 °C.

#### Microscopy

A coarse incision was milled directly into the surface of the high pressure frozen sample using the 30 kV: 16 nA FIB probe current in order to achieve a viewing channel for the SEM imaging. A window of about 50 μm in width was fine polished using the 30 kV: 600 pA FIB probe current. For acquisition of the data cube, a block face of about 50 μm width using the 600 pA FIB probe current and a slice thickness of 15 nm was defined. The data cube was acquired in a fully automated process. The FIB milling procedure was paused after each slice and the region of interest (ROI) on the block face was imaged using the 7.5 μm aperture in normal mode at 2.33 kV acceleration voltage. Images were acquired with both the in-lens and the back scattered electron (BSE) detectors. The lateral image pixel size was 7.5 nm, resulting in images of 7.72 μm width and 5.79 μm height. Line averaging (*N* = 256) and a scan speed of 100 ns dwell time were chosen for noise reduction. The cycle time for recording an individual image was 25.2 s. The milling time for removing each slice was 35.8 s. Accordingly, an image was recorded in about a minute. The final image series consisted of 40 individual slices, resulting in a volume of 7.72 × 5.79 × 0.6 μm^3^.

#### Image analyses

The obtained image sequences obtained using both the in-lens and the BSE detectors were aligned and segmented using the pixel classification workflow of the Ilastik software.^36^ The segmented stacks were imported into Drishti software to generate the 3D visualization. The magnetic particles were rendered using the BSE detector stacks, while the cellular membrane was rendered using the in-lens stack.

### (b) Microscopy

#### Sample preparation

*M*. *gryphiswaldense* cells of strain MSR-1 expressing mCherry–MamK were grown overnight to OD 0.2. One mL of the culture was washed by successive centrifugation for 10 min at 5 krpm and resuspension in PBS buffers. The final centrifugation was followed by a resuspension in 40 μL of low melt agarose at 35 °C. 15 μL of the agar suspension was sandwiched between a microscope slide and a coverslip placed between magnets and the sample was cooled for 10 min at 5 °C. This sample preparation ensured the alignment of the bacteria along the sample plane and allowed observation of live bacteria. For confocal fluorescence microscopy, the bacterial membrane was stained using FM 143 following supplier's recommendation for preparation.

#### Confocal fluorescence microscopy

Conventional fluorescence images were recorded using 488 nm and 561 nm laser line on a confocal microscope (SP5, Leica).

#### Super-resolution fluorescence microscopy

Photoactivated Localisation Microscopy (PALM) is a super-resolution microscopy technique that allows improving the spatial resolution of standard fluorescence microscopy by an order of magnitude.^37^ PALM is based on the single molecule detection and localization of photoswitchable fluorophores. By separating the emission of the fluorophores in time, it is possible to individually fit a Gaussian curve to localize with nm precision the molecules in a sample. A map of molecular coordinates of the fluorophores in the sample can be reconstructed with a precision of a few tens of nm. While for a typical PALM experiment a cell needs to be labeled with a photoswitchable fluorescent protein, it is also possible to use the photoblinking of some standard fluorescent proteins such as mCherry in a PALM-like experiment.

PALM was carried out on an Olympus IX-83 microscope equipped with 405 nm, 488 nm, 561 nm and 647 nm diode lasers and an oil immersion objective (Olympus OI 150×; NA1.45). Lasers are fiber coupled to the microscope and reflected to the sample using a quad band (405/488/561/635) dichroic filter. Emission was collected *via* a quad band (25 nm band pass 446/523/600/677) emission filter using a Hamamatsu Image-EM EM-CCD camera. Excitation of mCherry is achieved using the 561 nm line. In order to collect sufficient blinking events, sequences of 2000 images were recorded for each mapped area. Super-resolution images were generated using the Localizer plugin^38^ for Igor Pro (Wavemetrics).

## 3. Results and discussion

### (a) The magnetosome chain: paradigm of a 1D magnetic nanostructure?

The magnetotactic bacteria and their magnetosome chains are typically imaged using transmission electron microscopy (TEM). In TEM, the bacteria are typically prepared in such a way that they are dried on the surface of a carbon-film coated grid, and the imaging is the result of the projection of the object on a surface. Therefore, several artefacts can originate from the procedure. First, the bacteria, mostly made of organic matter, can be deformed due to drying, thereby possibly deforming the intracellular chain too. In addition, it is not clear how the chain is positioned with respect to the cell envelope, since magnetospirilla are helicoidally-shaped. Cryo-electron tomography (CET) has emerged as a powerful technique to avoid the artefacts listed above. In particular, the technique allowed the discovery of the magnetosome filament.^26,27^ However, several technical difficulties, including the so-called missing wedges, angles hardly accessible for imaging due to constraints in the electron microscope column, as well as contrast differences between the strongly contrasting magnetite nanoparticles and the poorly contrasting bacterial membrane associated with reconstruction problems, mean that the technique is still restricted to only a few laboratories worldwide.

**Fig. 3:**
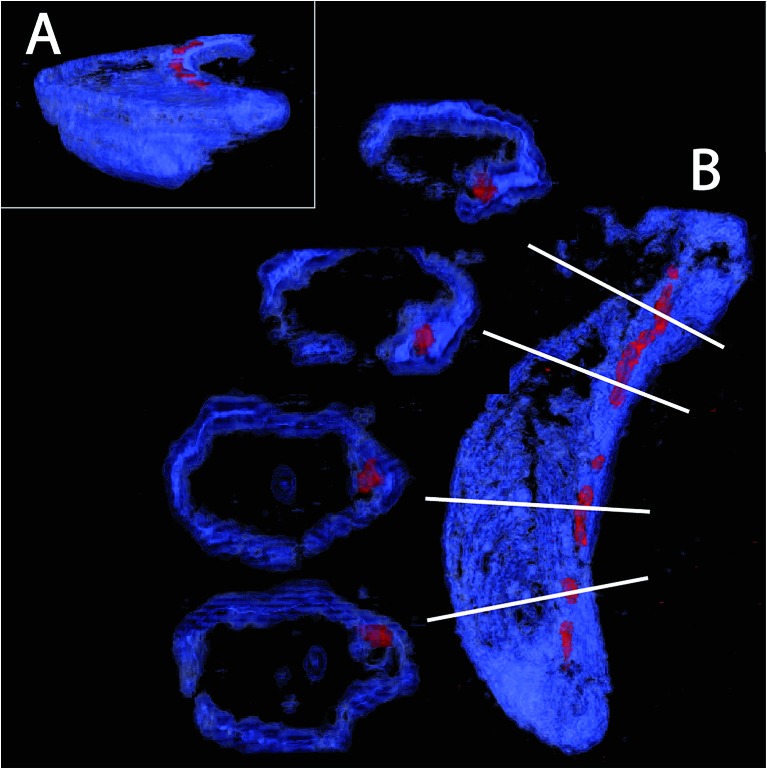
3D rendering of cryo-FIB sectioning and SEM imaging. The cell diameter is typically 500 nm to get an idea about characteristics length scales. A scale bar is not depicted here since the images are snapshots from a 3D rendering with perspective where therefore a pixel size can change as a function of the position on the image.

Here, we present the first images based on cryo-FIB sectioning of cryogenic fixed magnetotactic bacteria MSR-1 cells imaged by SEM (Fig. 3, ESI video 1†). The 3D rendering of a cross section of the cell with the top part being “deleted” by a clipping plane shows that the magnetosomes (particles in red) follow the inner curvature of the cell (cell membrane in blue), resulting in a slightly bent chain (Fig. 3A). Magnetosomes, in addition, seem to always be present in close proximity to the membrane (Fig. 3B). Magnetosome invaginations were originally shown to be present in AMB-1 (ref. 26) and later in MSR-1, but in the latter it is not clear if this invagination phase is transient or permanent.^39^ The fact that both strains are helicoidally-shaped makes the positioning of the chain along the membrane a very difficult 3D problem: the chain would have to be positioned as the axis of a screw would be, with the membrane in constant contact around it. In addition, if the magnetosomes are continuously attached to the inner membrane, the role of the magnetosome filament as a mechanical stabilizer of the chain can be questioned.

### (b) Assembly of magnetosomes

**Fig. 4:**
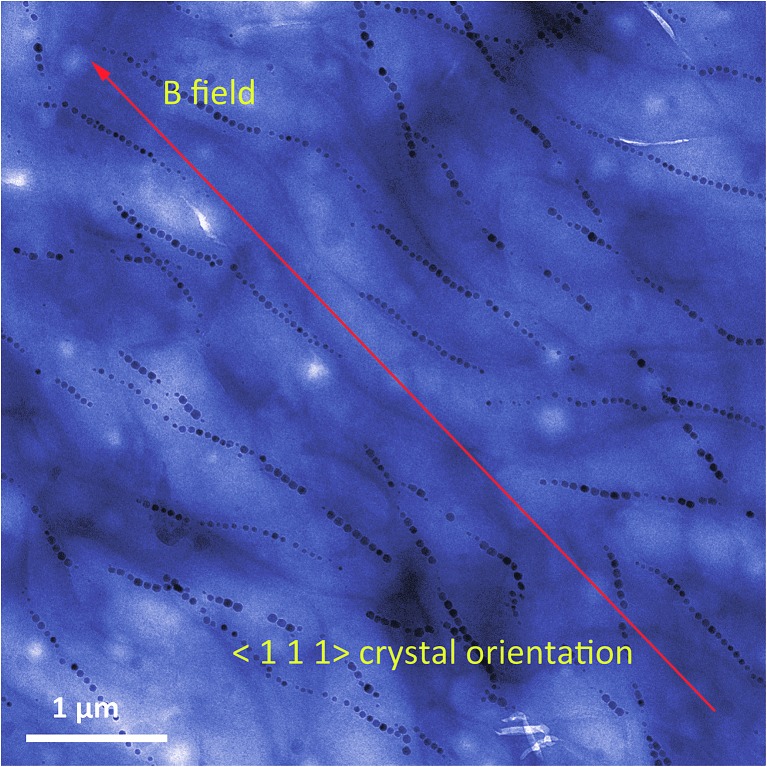
False colour transmission electron microscopy image of aligned magnetotactic bacteria. The bacteria are aligned on the TEM grid by the application of a strong external magnetic field. For *Magnetospirillum gryphiswaldense*, the magnetosome crystals are oriented along the 〈1 1 1〉 crystallographic direction, as depicted on the image. This corresponds to the easy axis of magnetization for isotropic magnetite nanoparticles.^19^

As explained above, the magnetosome chain is hierarchically structured. It is formed as the result of the assembly of the individual magnetosomes. In the chain, all the magnetosomes are aligned along the same crystallographic axis, which corresponds to the easy axis of magnetization (Fig. 4).^19^

**Fig. 5:**
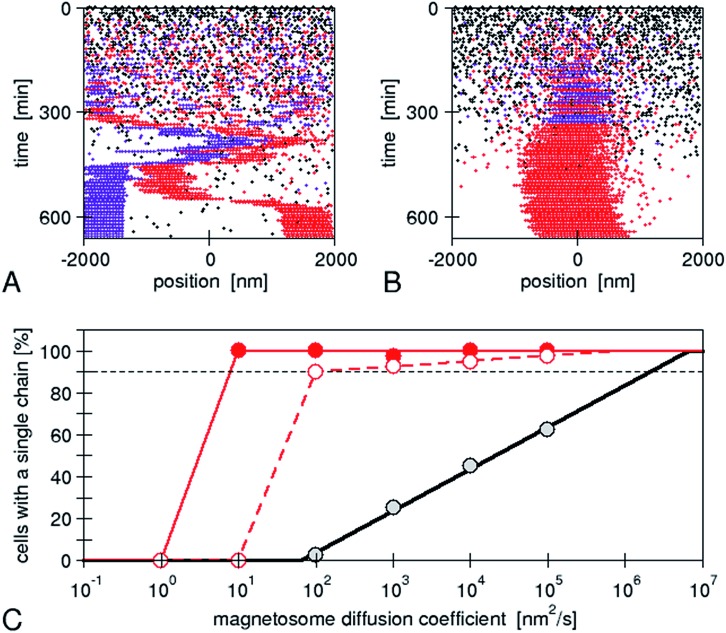
Simulations of *de novo* magnetosome chain formation with and without active transport of magnetosomes: simulation trajectories for diffusing magnetosomes (A) and magnetosomes that are actively transported toward the centre of the cell (B). In both cases, open black circles show empty magnetosome vesicles, and red and violet circles show magnetosomes containing a crystal with negative and positive orientation of the magnetic moment, respectively. The simulations were carried out with the same set of parameters as in ref. 22 and a magnetosome mobility characterized by a diffusion coefficient of 10^4^ nm^2^ s^–1^ and an active driving force *F*_act_ = 0.01 pN (in B). (C) Fraction of simulations that result in a cell with a single chain, as a function of the magnetosome mobility (diffusion coefficient). A gradual increase is obtained for the case of diffusive motion (black circles), while a threshold behaviour is seen for active transport (with *F*_act_ = 0.1 and 0.01 pN for the solid red and open red circles, respectively). The lines extrapolate these simulation data (from ref. 22) to a larger range of mobilities.

The assembly is performed by the interactions between physical and biological forces. We recently introduced a model to theoretically study the interplay of these forces.^22^ We created “*in silico* mutants”, defective not in individual genes, but in entire physical processes, for example, mutants lacking magnetic interactions. Using the model, we have shown that a purely physical process, *i.e.* the diffusion of the magnetosomes “biased” by their magnetic interactions, does not reliably result in a chain pattern, rather two or even more short chains are observed, typically with different magnetic polarization (Fig. 5), a behaviour later observed in another bacterial strain experimentally.^40^ Therefore, we concluded that a mechanism of active transport of magnetosomes to the cell centre is required for reliable chain formation. The driving force of such active transport needs to exceed a threshold, which we showed to be easily accessible for a cytoskeletal machinery such as those possibly expected for *mamK*.^22^ Thus, specific biological forces play a critical role in the magnetosome chain assembly. We note however that directed transport might not be absolutely required for chain assembly, but appears to be necessary for the reliable chain assembly in bacterial cells. Indeed, in our simulations, we varied the mobility of the magnetosomes over a wide range (with the mobility of large proteins in bacterial cytoplasm as an upper limit) and observed that chain formation becomes more robust with increasing mobility. By extrapolating our simulation results (Fig. 5), we can predict that a diffusion coefficient of 2 μm^2^ s^–1^ would be needed for reliable formation of a single chain (in more than 90% of the cells as observed experimentally^40^). Such a high diffusion coefficient might be achieved in an aqueous solution, but is highly unlikely in cytoplasm, where large proteins (with radius 2–5 nm) have diffusion coefficients comparable to this value (1–10 μm^2^ s^–1^ in bacterial cytoplasm^41,42^). Due to the strong size-dependence of diffusion in cytoplasm,^42^ diffusion of magnetosomes should be considerably slower, even more so if the magnetosomes are indeed attached to the inner membrane as invaginations. Thus, in a cell, such rapid diffusion could only be achieved through active processes that enhance diffusion through active (energy-dependent) but random motion (“active diffusion”), a mechanism known for cytoskeletal transport.^43^ This observation indicates that an important constraint/limitation to chain assembly in the cell is the low mobility of large objects such as magnetosomes in the cytoplasm. While active diffusion provides a possible explanation for chain assembly in the cells, there is no direct evidence for such motion. Moreover directed active transport provides the additional benefit of functioning as a mechanism for localizing the chain in the cell centre, and for controlled repositioning of the chain after cell division.^44^ In either case, the active processes are likely based on the magnetosome filament, which possibly acts as a polymerization or depolymerization motor.

We have therefore studied the role of biological determinants in more detail here. One of the functions of protein networks *in vivo* is to provide cells with mechanical properties. For instance, actin is a protein that forms filaments in eukaryotic cells and is used for tasks requiring mechanical forces such as cell motility, maintenance of cell shape and organelle organisation.^45^ Because such filaments are too thin to provide contrast by light absorption using regular optical microscopy, researchers image them *in vivo* using fluorescence microscopy and a fluorescence marker. The markers allow us to detect the presence and map the network of the stained proteins, with a maximum resolution given by the diffraction limit theorem, *ca.* 250 nm in a typical experiment. In some instances, this resolution is not sufficient to gain insight into the protein network properties. As mentioned above, 2 particular proteins MamJ and MamK have been studied so far, specifically in the magnetospirilla *Magnetospirillum magneticum* and *Magnetospirillum gryphiswaldense*. It has remained unclear if the phenotype observed for the deletion strains *i.e.* the “observable” characteristics differentiating the mutant from the unmodified “wild-type” cells is directly due to the non-expression of this particular gene and of the resulting non-expression of the associated protein, or if this results from an indirect role associated with the process where *e.g.* other protein expression levels are modified as well. For example, in AMB-1, the roles of *mamJ* and *limJ* are redundant, showing that potentially in the absence of the one, the other gene present in the machinery of the cell is capable of taking over and therefore also potentially blurring the role played by a giving gene in the organism.^24^ The same redundancy has been observed for *mamK* with the presence of another gene, *mamK-like*, that is indeed capable of forming filaments in the *mamK* deletion mutant in AMB-1 cells.^32^

**Fig. 6:**
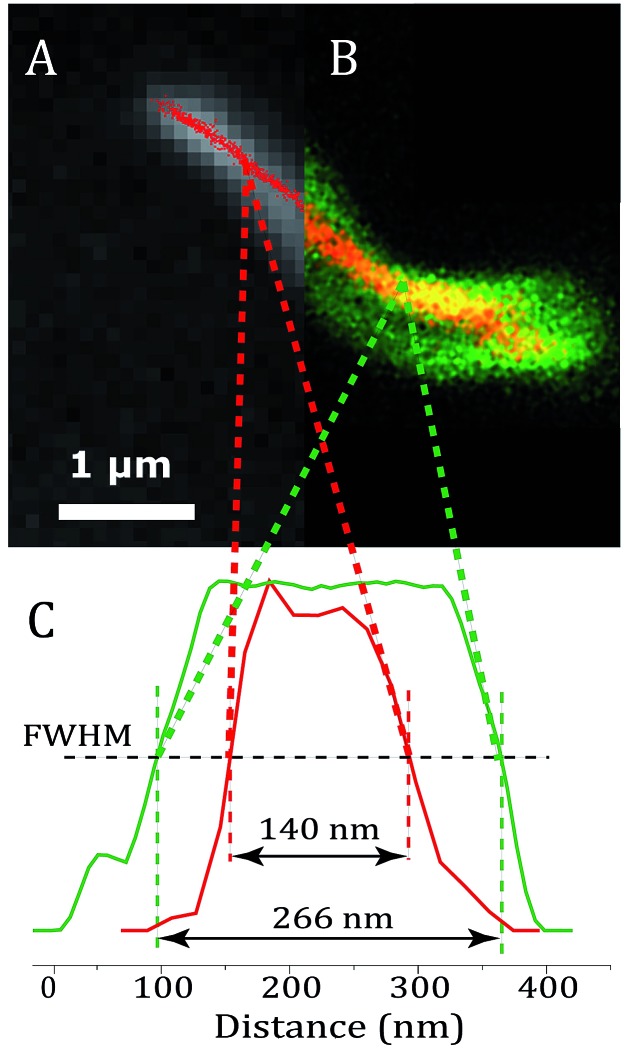
(a) Super-resolution image of MamK–mCherry filaments (red dots) in a living bacterium; (b) an equivalent confocal fluorescence image of MamK–mCherry filaments (yellow signal) in a living bacterium, the membrane of which is dyed using FM-143 (green signal). (c) Profile of the fluorescence signal of mCherry extracted from the super-resolution image (red) and from the confocal image (green).

Fig. 6 shows an image of an mCherry–MamK filament *in vivo* recorded using confocal fluorescence microscopy (Fig. 6b) in comparison with a super-resolution image (Fig. 6a). As can be observed on the extracted profiles (Fig. 6c), the extent to which the filament can be located in the cell in the confocal image is limited by the diffraction limit of the optical system, which is slightly better than half the width of the bacterium. In the super-resolution image, each red dot corresponds to the calculated position of an mCherry molecule that has undergone a blinking event. The standard deviation in the determined position of each of these molecules was calculated to be an average of 22 nm across all localized emitters. In contrast, the full-width at half maximum intensity of the filament, show in Fig. 6, is 140 ± 32 nm, much larger than our localization precision, clearly indicating the presence of filaments comprised of supramolecular bundles of proteins in these organisms.

**Fig. 7:**
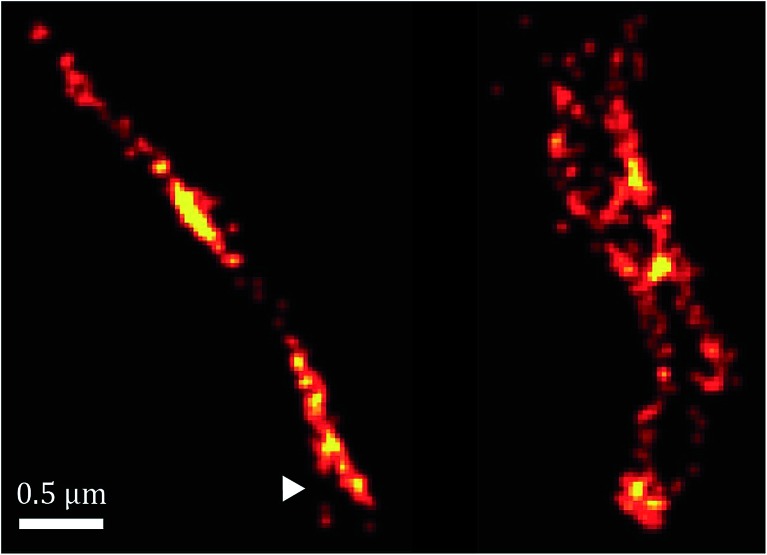
Super-resolution images of the MamK filament *in vivo* showing branching (white triangle).

**Fig. 8:**
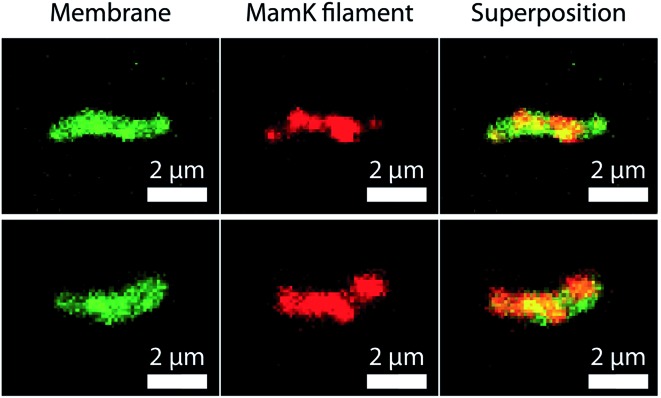
Confocal fluorescence images of MSR1 MamK–mCherry stained with a membrane dye (FM-143). The first column shows the fluorescence of the membrane, the second the fluorescence of the MamK filament and the third column is a superposition of the images of the first two columns. The spatial distribution of MamK suggests that the filaments form clusters.

Furthermore, as shown in Fig. 7, the spatial distribution of MamK in MSR-1 does not typically correspond to the expected pattern of a single filamentous structure extending from pole to pole of the cell. Indeed, some bacteria exhibit branching of the MamK filament or a heterogeneous (clustered) distribution of the MamK–mCherry construct across the cell. This observation made us revisit our confocal imaging data in more detail, and we indeed find that a majority of bacteria exhibit clustered MamK (Fig. 8).

The data above show that the distribution of MamK is more complex than that previously thought, and highlight the need for more detailed high-resolution imaging. However, it is worth pointing out that our fluorescence microscopy data does not necessarily suggest that the magnetosome filament is not straight, and it might simply be a consequence of the dynamic localization of the proteins that finally assemble as bundles along the magnetosome chain to provide the commonly accepted template for the mechanical stabilization of the magnetosome chain.

## 4. Conclusions

In conclusion, we have seen that magnetotactic bacteria are able to produce magnetosomes and align them in a chain that is nearly perfectly 1D. Further work will however be necessary to determine the organization of the magnetosomes in 3D, in the case of the most studied spirilla, but also for other bacterial morphology and as a function of magnetosome organization, since some strains produce more than one chain per bacterium. Cryo-FIB may become a powerful tool to advance this problem, but will remain impeded by the time consuming nature of current electron tomographic techniques.

In addition, if the established MamJ–MamK system is also very appealing because of its simplicity and apparent widespread application, we recall here that the MamJ protein is not universally conserved, but absent from many magnetotactic bacteria where chains are observed. Therefore an alternative scenario must be considered. Furthermore, it was shown that the phenotype associated with *mamK* deletion mutant in MSR-1 and AMB-1 is not fully consistent with the presented model, since clusters of magnetosomes would be expected if those would be free to move. This is a further hint towards a binding of the magnetosomes, not only to MamK filaments but also to another structure, which could be the inner membrane as shown in Fig. 3, or another structure to be identified.

Here we also show that the MamK filaments are not only found next to the magnetosome chain, but that its intracellular localization is much more dispersed than initially described/thought, possibly partly due to reduced resolution associated with optical microscopy techniques. This could be interpreted as a further indication that MamK is not only the protein at the origin of the materials building the magnetosome filament, but might also be involved in the directed transport of the magnetosomes, as suggested by our simulations and previous experimental work.^44^

Still, we are convinced that the magnetotactic bacteria represent an interesting model for a possible way of forming a functional magnetic anisotropic structure serving as an actuator. Such actuators are difficult to form synthetically but would be of interest for numerous applications, and therefore there is still much to learn from these microorganisms, which are more intricate than initially thought.
